# Experience with a Multinational, Secondary School Education Module with a Focus on Prevention of Virus Infections

**DOI:** 10.4269/ajtmh.16-0661

**Published:** 2017-04-24

**Authors:** Laura Doornekamp, Karen M. Stegers-Jager, Odette M. Vlek, Tanja Klop, Marco Goeijenbier, Eric C. M. van Gorp

**Affiliations:** 1 Department of Viroscience, Erasmus MC, University Medical Center Rotterdam, Rotterdam, The Netherlands; 2 Institute of Medical Education Research Rotterdam, Erasmus MC, University Medical Center Rotterdam, Rotterdam, The Netherlands; 3 Ruigrok NetPanel, Amsterdam, The Netherlands; 4 Science Center Delft, University of Technology Delft, Delft, The Netherlands

## Abstract

Worldwide, virus infections are responsible for many diseases in terms of morbidity and mortality. Vaccinations and therapies are only available for relatively few virus infections and not always where they are needed. However, knowledge of transmission routes can prevent virus infection. In the context of this study, we measured the effects of a secondary school education module, named Viruskenner, on knowledge, attitude, and risk behavior as these relate to virus infections. A nonrandomized intervention study was conducted between April and August 2015 to assess the effect of this 2-month education module on knowledge, attitude, and behavior of 684 secondary school students in the Netherlands, Suriname, and Indonesia. For the Netherlands, a control group of a further 184 students was added. Factor analysis was performed on questions pertaining to attitude and behavior. Comparative analyses between pre- and posttest per country were done using multiple linear regression, independent sample T-tests, and one-way analysis of variance. These showed a significant increase in knowledge about virus infections and the prevention of infectious diseases among the Dutch and Surinamese groups, whereas a trend of increased knowledge was evident among the Indonesian participants. The Dutch control group showed an overall decrease in knowledge. Regression analyses showed that there was a significant interaction effect between participation and time on knowledge, attitude, and awareness and behavior and risk infection. Attitudes improved significantly in the intervention group. Pearson correlation coefficients between knowledge, attitude, and behavior were found to be positive.

## Introduction

Viruses are responsible for almost half of all emerging infections worldwide and are among the most emerging pathogens.^1^^–^^3^ Most virus infections are not treatable with antivirals and neither are they preventable with vaccines. Therefore, education plays a key role in raising awareness for infectious diseases and preventing the spread of virus infections.^4^ A population that is aware of the different ways a virus can be transmitted and does know how to embed effective preventive methods in daily life can protect themselves against virus infections. This principle is based on the knowledge, attitude, behavior (KAB) model, showing that increased knowledge can change people's attitudes and lead to behavioral change.^5^^–^^7^

A foundation for health-related attitudes and behavior is laid in early stages of life. Following many theories, the likelihood of changing attitude is high in adolescence.^8^ Also, the World Health Organization (WHO) states with their Health Promoting School Framework that schools are a good environment to start promoting health.^9^ Five years ago, a consortium of scientists and teachers developed a school-based education module named Viruskenner, regarding virus infections. This module aimed to teach students how to prevent virus infections. The module started with one secondary school in the Netherlands and evolved to a project with eight different secondary schools in the Netherlands. In 2014, the first Surinamese school joined and in 2015 the first Indonesian school. These two countries were already involved with the organizing institute by an international collaboration on emerging infectious disease population studies, facilitating easy communication and logistics. The Viruskenner module was extensively evaluated by independent researchers in the early years of the project. The conclusions of these evaluations led to improvement of the module and the questionnaires used. For example, in 2012, the concept of students being coached by an infectious disease expert was introduced. When becoming an international education module, it was interesting to see the impact of Viruskenner in different countries on knowledge, attitudes, and behavior as they relate to virus infections, and find out which educational factors play a role in these changes.

Educational programs that address infectious diseases are quite common, although most education is focused on a specific infection or a group of infections, particularly human immunodeficiency virus (HIV) and other sexually transmitted infections. A recent systematic review and meta-analysis evaluated 64 school-based sex education programs in middle- and low-income countries. Most of these programs (55 of 64) focused on comprehensive sex education, with the remaining nine focusing on sexual abstinence. About half of the studies (33) were included in the meta-analysis and showed an overall positive effect on HIV-related knowledge, condom use, the initiation of sexual intercourse, the number of sexual partners, and self-efficacy.^10^

Although HIV is among the virus infections that place the highest burden on society, it is not the only virus that significantly impacts global health. Besides HIV, lower respiratory tract infections (e.g., influenza), and diarrheal diseases (like norovirus) also belong to the 10 leading contributors to the global burden of disease.^11^ Furthermore, arthropod-borne diseases (like dengue) have a very high incidence.^12^ Remarkably, virus infections other than HIV are less frequently addressed in education modules. For example, only a few trials were carried out to measure the impact of an educational intervention for viral hepatitis, human papillomavirus (HPV), dengue, and influenza.^13^^–^^17^

Most of the educational interventions that were analyzed showed positive results in improving knowledge and attitude pertaining to the subject of the intervention. Given the success of education programs about HIV, education modules about other virus infections that have global impact might also work. We developed an education module that focuses on multiple viruses with different transmission routes and all with global impact: HIV, dengue, hantavirus, chikungunya, Middle East respiratory syndrome (MERS) coronavirus, HPV, norovirus, viral hepatitis, measles, and influenza. We studied the efficacy and success of the education module in three countries, Netherlands, Suriname, and Indonesia, each differing in culture, circulating viruses, and infection pressure. The education module aims to effectively increase knowledge, attitudes, and behavior regarding several virus infections in each of these different circumstances.

## Materials and Methods

### Participants and setting.

Schools in three countries participated in this nonrandomized intervention study: four in the Netherlands, a high-income country in Europe; one in Suriname, an upper middle–income country in Latin-America; and one in Indonesia, a lower middle income country in southeast Asia.^18^ The effect of the education module was measured per country, by comparing the results of a pre- and posttest. The situations per country, for example, culture and school system, were too different to make a fair comparison between countries. However, the target group for the education module is the same in each country and the concept of the module and measurements were as comparable as possible.

Secondary schools in the Netherlands, Suriname, and Indonesia had been invited to apply to participate in the education module with their 10th grade students (generally 14 or 15 years of age). All schools were well-known public schools for students with an above-average socioeconomic status. The school in Suriname that participated had about 840 students and was located in Paramaribo, the capital and largest city of the country in inhabitants. The Indonesian school that participated was located in Surabaya, the second largest city in the country. This was a senior high school (grade 10–12) and had about 1,200 students. The four schools in the Netherlands ranged in number of students from 1,600 to 2,400 and were from different regions but all in the Dutch urban agglomeration, including one school from Amsterdam, the capital city of the Netherlands.

The 10th grade is the final stage of the junior high school in the Netherlands, which means that all students have, until then, followed the same subjects and have expressed their interest in the choice for a special curriculum. For example, a beta scientific curriculum, which includes the following subjects: biology, physics, chemistry, and mathematics.

In the Netherlands, the schools that were invited to participate were all schools that offer students an option for Technasium, which is an elective course for students interested in beta scientific subjects.^19^ The participating students had all chosen this special curriculum with additional technical courses. Information about the module was disseminated via the project website and the Technasium network coordinator. A control group for the Dutch intervention group was selected at one of the participating Dutch schools. Thus, although they had not opted for the Technasium curriculum, they do have a similar background and social environment. School curricula are defined differently in each country. In the Netherlands, students choose a profile and we defined “nature and science” and “nature and health” as scientific profiles. In Suriname, students can choose biology, and we defined this as a scientific profile. Indonesian students can choose between a social profile (Ilmu Pengetahuan Sosial [IPS]) and a science profile (Ilmu Pengetahuan Alam [IPA]). We defined IPA as a scientific profile. In both Suriname and Indonesia, schools that matched most closely, in terms of grade and education level, with the Dutch intervention group were invited to participate in the study. Of the Dutch schools, two were preuniversity education level (known in the Netherlands as “VWO”) and two were mixed preuniversity education level and advanced general secondary education (known in the Netherlands as “HAVO”). The Surinamese participants were from one VWO school, which is comparable with the Dutch VWO education level. These Surinamese participants can therefore be seen as preuniversity education level.^20^ The Indonesian participants were from one Sekolah Menengah Atas (SMA) (high school), which is comparable with HAVO in the Netherlands and internationally known as advanced general secondary education.^21^ The Dutch control group consisted of students with preuniversity education level and advanced general secondary education level.

### Design of the intervention.

The Viruskenner education module is based on the “learning-by-doing” principle. Students are challenged to create a prevention tool for a specific virus infection. By involving students in real-life science-based problems and stimulating active learning (searching for information, test possible solutions and present their idea) a high impact can be achieved.^22^^–^^24^ In each country, the 2-month module started with a national opening day, during which all participants of that country were introduced to the field of infectious diseases and viruses by means of four short lectures from experts in the field of virology, public health, and infectious diseases. An optimal learning effect can be reached by bringing students in contact with experts.^25^ So, in all countries one or two Dutch experts from the department of Viroscience in the Erasmus Medical Center in Rotterdam were assigned to a class to coach them during the project. The students were supposed to work in groups of four to six students in competition with the other groups.^22^^,^^26^ Each group worked on one of the viruses of the subject list including HIV, dengue, hantavirus, chikungunya, MERS coronavirus, HPV, norovirus, viral hepatitis, measles, and influenza. Students developed a prevention tool to disseminate this knowledge among their peers and, in doing so, help prevent virus infections that impact local or global health. During the three national final days (one in each country), the best groups per class, selected by the teachers and coach during a school final, presented their results and final product to their peer students and a jury. This independent jury was selected per country and based on proven expertise in virology, communication strategy, and/or overall creativity. In each country, the jury chose two winners: the most informative presented prevention tool and the most creative prevention tool.

**Figure 1.**
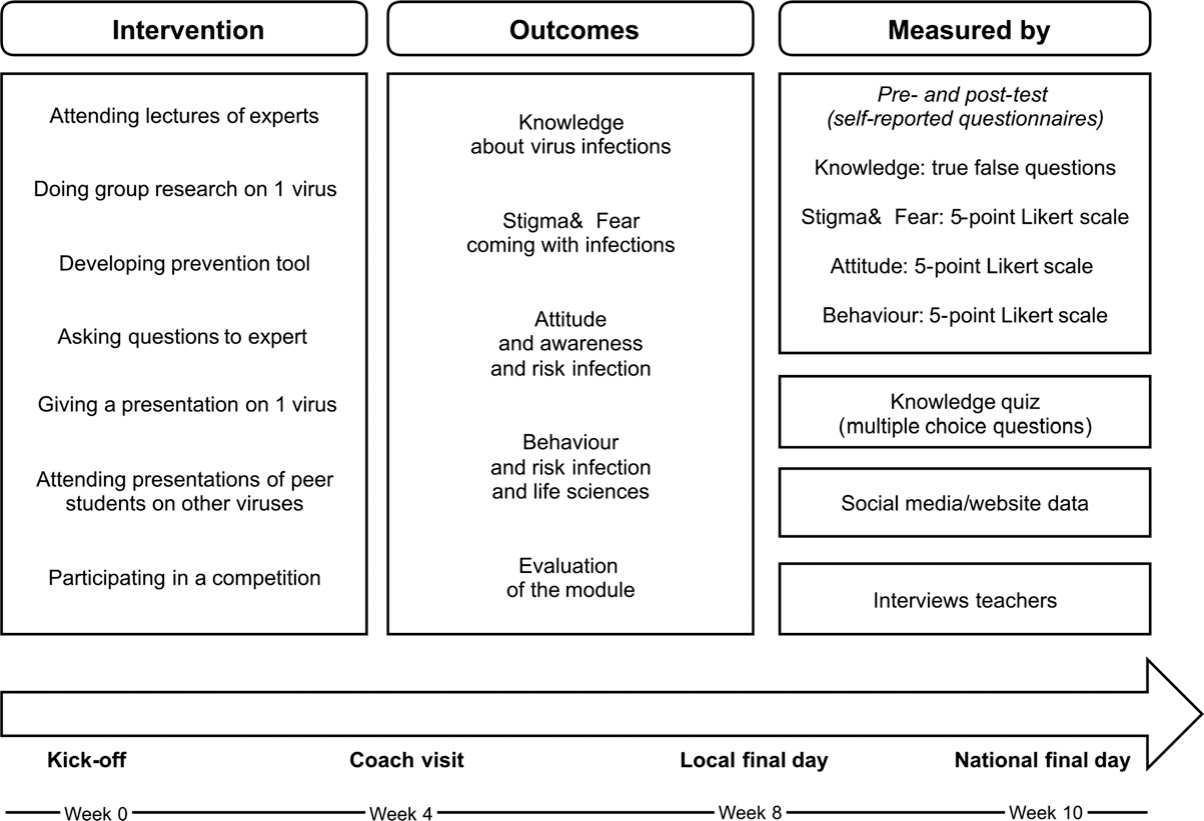
Overview of all elements of the intervention and the outcomes that are measured by different instruments. The timeline shows the most important events during the project.

The study was conducted between April and August 2015. A pretest was performed 1 or 2 days before the start of the module to assess their basic knowledge, attitude, and behavior; a posttest 5–7 days after its completion to let the information settle in their memory and give the students some time to evaluate their attitude and behavior a few days after the final day. Other measurement instruments were used to get additional information (Figure 1

).

During the intervention, students could use the modules' website (www.viruskenner.nl) and other supportive resources, like a YouTube channel and a Facebook page (all in Dutch and English and available for all participating countries), to find more information on the project and on virus infections and to disseminate information about their prevention tools.^27^^–^^29^

### Instruments.

The effect of the education module was measured by the KAB model. Given that there was no validated instrument to assess knowledge, attitude, and behavior regarding several viral infections, we used a self-designed questionnaire (Supplemental Table 1). The questionnaire was based on 5 years' experience with the education module. A team comprising two senior virologists, a communication scientist, and an education expert developed the questionnaire, which was refined after a pilot with a group of 60 students from a school similar to the participating schools. The questionnaires used for the Dutch and Surinamese schools were in Dutch. The questionnaires for the Indonesian students were first translated into English by a Dutch researcher and then into Indonesian by a native speaker of the language.

The pre- and posttest questionnaires addressed five areas: 1) sociodemographic factors, 2) stigma and fear, 3) attitude and behavior, 4) knowledge on viruses and infectious diseases in general, and 5) the opportunity to write down questions or comments about the questionnaire or module. The posttest had an additional category—6) perceptions of the project.

A principal component analysis (PCA) with varimax rotation was performed for the attitude and behavioral questions on the results of the pretest questionnaires, as suggested in literature.^30^ Varimax was the preferred rotation because this results in a small number of factors per variable and a small number of variables per factor. This is the most popular type of rotation because it makes the interpretation of the data more reliable and easier.^31^ One of the behavior items (“I do not use a condom when I have sexual intercourse”) was excluded because of more than 10% missing values. The remaining missing values were randomly spread over the sample population. The sample size was big enough to delete these cases list wise. The Kaiser–Meyer–Olkin (KMO) value is a statistic that measures how much two random variables correlate. A KMO value greater than 0.8 represents a small partial correlation which makes a factor analysis more useful. In this study, the KMO value was 0.849, which means there were relatively compact patterns of correlations and the factor analysis would provide reliable components.^32^ The number of extracted factors was based on the objective and interpretability criteria mentioned in Schönrock–Adema and others: 1) the screen test, 2) eigenvalues > 1.5, 3) > 5% of the variance explained by all factors, and 4) interpretability. However, the criterion of eigenvalue > 1.5 led to only two components, which was not interpretable. Therefore, we set the norm of an eigenvalue back to greater than one (Kaiser's criterion).^33^^,^^34^ The PCA with varimax rotation finally resulted in four components. The reliability per component was calculated by Cronbach's alpha (Table 1

**Table 1:** Attitudes and behavior

Component	Construct	Number of items	Example item	Cronbach's alpha
1	Attitude and awareness	3	I think it is important to know about viruses.	0.797
2	Attitude and risk infection	5	I think getting a tattoo is a risk.	0.572
3	Behavior and risk infection	4	I protect myself against mosquito bites when I go to a tropical country/into the forest.	0.491
4	Behavior and life sciences	5	I watch science programs or documentaries on TV.	0.725

). Internal consistency for the components “attitude and awareness” and “behavior and life science” was above 0.7 and therefore acceptable. The components “attitude and risk infections” and “behavior and risk infection” should be interpreted with caution, because of the diversity of the constructs.^35^

An additional instrument to measure knowledge was a live multiple-choice quiz, which was implemented at the end of the final day. In the Netherlands and Suriname, portable electronic devices (keypad and software from Interactive Voting System^®^) were used by the students to answer 40 knowledge questions. In Indonesia, these portable electronic devices were not available, so the knowledge quiz was done by voting with colored papers; therefore, recording these results was not possible.

To obtain more information about factors that influenced the impact of the education module, teachers of all four participating schools in the Netherlands were interviewed when they had completed the education module. The aim of this additional qualitative component was to determine possible confounders which might have influenced the difference in outcomes between the pre- and posttests and to find out whether the teachers noticed increased knowledge or improved attitude and/or behavior among their students. Although the teacher interviews were carried out in the Netherlands only, the module was evaluated in each country. In Suriname and Indonesia, the project was evaluated with the local organizing teams but not per individual teacher. In the Indonesian and Surinamese culture, hierarchy is strong and extensive evaluation uncommon. Therefore, the teachers preferred a general evaluation with the head of the school. However, we do feel these interviews were less helpful because the heads of the schools were not closely involved in the project. The Dutch teacher interviews were semistructured and took about 30 minutes each. Questions that were asked included “how was the contact with the coaches?” and “what have the students learned during the project?” Teachers were interviewed in their classrooms after the classes had filled out the posttest questionnaire.

Finally, user data from the website and social media were analyzed after the completion of the module to find out which supportive resources were most popular during the education module (Supplemental Figure 1).

### Outcomes.

The primary outcomes in this study were knowledge, attitude, and behavior and stigma and fear. Stigma and fear, attitude, behavior were measured on a 5-point Likert scale. All these outcomes ranged from 1 (strongly disagree) to 5 (strongly agree). Stigma and fear were measured with two questions and mean values were calculated. The outcome “stigma” was used in this study to describe a negative thought regarding people with a HIV infection. The stigma was expected to be high before the module started. By gaining knowledge, the stigma could be decreased. The outcome “fear” in this study aims to measure how afraid people are to get infected in case of a large outbreak; at the time of this study, Ebola was the best example. The attitude and behavior questions were subdivided into four components by the factor analysis and the mean score per component was calculated. The first component was attitude and awareness and this component represents how aware students are of virus infections and how important they consider them. The second component was attitude and risk infection and this one shows how students evaluate the risk for getting an infection. The third component, behavior and risk infection, showed what students would do in case of a risk for infection. The fourth and last component of the factor analysis was behavior and life sciences and represented what students do to gain information about viruses and related science (Table 1).

Unstandardized coefficients (B) as outcomes of the regression analysis showed which factors contribute significantly to these four attitude and behavior components and to knowledge.

The outcome knowledge represented the student's knowledge regarding infectious diseases in general and the viruses in specific that were included in the education module. Knowledge was measured in the questionnaire by means of the responses to 32 questions, which had to be answered with “true” or “false.” Each correct answer resulted in one point, an incorrect answer in zero points. The mean percentage of all knowledge questions that were answered correctly was calculated per group and ranged from 0% to 100%. Knowledge outcomes from the quiz were calculated in percentages. As a secondary endpoint, the perceptions of the students about their participation in the project were evaluated. Ten statements measured if students enjoyed working on the project and if they thought the project was informative. This was scored on a 5-point Likert scale and ranged from 1 (strongly disagree) to 5 (strongly agree).

### Data management and analysis.

The questionnaires were read by the open source optical mark recognition program SDAPS (Benjamin Berg, Karlsruhe). Correct reading was checked manually by two different persons. All data were imported into one database and analyzed with IBM SPSS version 21. All questionnaires in which less than 90% of the knowledge questions had been answered were excluded. Cases that showed a variance equal to zero in the Likert scale questions were excluded for analysis on these outcomes. Items with more than 10% missing values were deleted. In all analyses, *P* < 0.05 was considered significant.

Descriptive analyses were performed to calculate the frequencies of students' characteristics and Pearson's χ^2^ test was used to identify significant differences between the characteristics of the groups in the pre- and posttest. Correlations between knowledge, attitude, and behavior were calculated for all students in the intervention group, with a Pearson's coefficient.

The average knowledge per country in the pre- and posttest situations was compared by an independent sample T-test. Effect sizes (ES) were calculated with Cohen's d. Effect sizes greater than or equal to 0.30 were considered medium, and those greater than or equal to 0.50 as large.^36^

Multiple linear regression analysis was used to find factors that influenced the knowledge, attitude, and behavior outcomes. Time point (pre- and posttest) and participation (intervention and control group) and the interaction between these two variables were added as independent variables, as well as gender, age, education level, school, and country. Tolerance values were computed to assess multicollinearity. Values below 0.2 were viewed as potentially problematic.^30^

Stigma and fear were compared between pre- and posttest with a one-way analysis of variance (ANOVA). The sample size allowed us to calculate differences between the components of the factor analysis in pre- and posttest per country with a one-way ANOVA test. Perceptions of the project were measured only after the module had finished. Means and standard deviation were summarized per country. The data set is provided in the supplementary materials.

### Ethics.

The study was carried out in accordance with the Declaration of Helsinki. According to Dutch law, this study was exempt from medical ethical approval requirements. The Technasium Network in the Netherlands approved this study to be performed at the Dutch Technasium schools and informed the students and parents. In Suriname and Indonesia, the headmasters of the schools approved conducting the Viruskenner module and evaluations at their schools and informed the students and their parents. Participation was voluntary and anonymity was guaranteed.

## Results

### Participants and setting.

**Figure 2.**
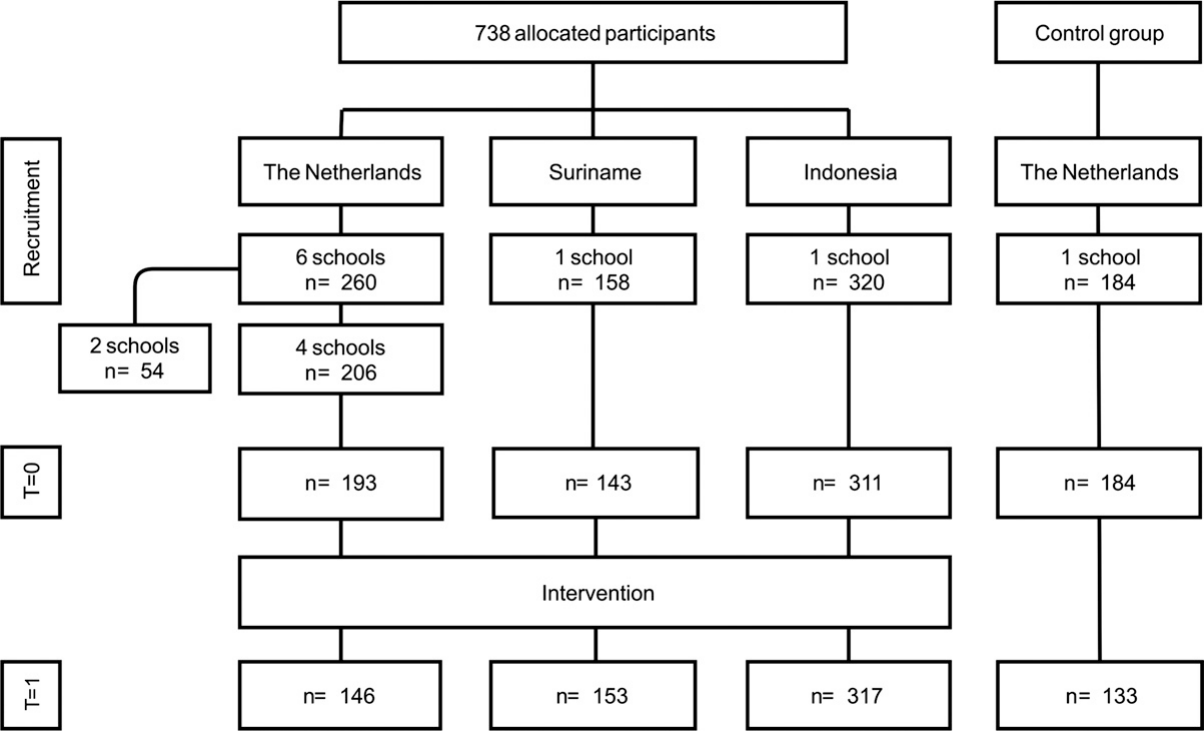
Study flowchart. T = 0 represents the pretest and T = 1 represents the posttest after 10 weeks.

In 2015, a total of 684 (of 738) secondary school students participated in the Viruskenner education module. Two of the participating schools in the Netherlands, dropped out (54 of 260 Dutch students, representing 20.7% of them) because of noncompletion of the module and evaluation program. One school dropped out because the teacher got sick after the kick-off and the other school could not attend the final day because it clashed with another school activity that day (Figure 2

). In Suriname, 158 students participated and there was no dropout. This was also the case in Indonesia, where all 320 students completed the education module.

Response rates for the Netherlands were 95.6% of participants for the pretest and 70.9% for the posttest. In Suriname, these percentages were 90.5 and 96.8%, respectively, and for Indonesia 97.2 and 99.1%, respectively. The control group had a response rate of 100% for the pretest and 73.4% for the posttest.

Table 2

**Table 2:** Students' characteristics

	Dutch intervention group			Dutch control group			Suriname			Indonesia		
Time point	T = 0	T = 1	p (χ^2^)	T = 0	T = 1	p (χ^2^)	T = 0	T = 1	p (χ^2^)	T = 0	T = 1	p (χ^2^)
Gender (%)	193 (100)	144 (100)	0.535	182 (100)	133 (100)	0.955	142 (100)	153 (100)	0.718	308 (100)	317 (100)	0.825
Boy	140 (72.5)	100 (69.4)		95 (52.2)	69 (51.9)		62 (43.7)	70 (45.8)		129 (41.9)	130 (41.0)	
Girl	53 (27.5)	44 (30.6)		87 (47.8)	64 (48.1)		80 (56.3)	83 (54.2)		179 (58.1)	187 (59.0)	
Age (%)	193 (100)	145 (100)	0.040	183 (100)	132 (100)	< 0.001	143 (100)	153 (100)	0.693	311 (100)	316 (100)	< 0.001
< 14	–	–		2 (0.0)	3 (0.0)		–	–		–	–	
14	100 (51.8)	55 (37.9)		78 (42.6)	27 (20.5)		1 (0.0)	0 (0.0)		4 (0.0)	2 (0.0)	
15	92 (47.7)	89 (61.4)		97 (53.0)	89 (67.4)		22 (15.4)	20 (13.1)		129 (41.5)	64 (20.3)	
16	1 (0.0)	1 (0.0)		6 (0.1)	13 (9.9)		52 (36.4)	59 (38.6)		175 (56.3)	233 (73.7)	
> 16	0 (0.0)	0 (0.0)		0 (0.0)	0 (0.0)		68 (47.6)	74 (48.4)		3 (0.0)	17 (0.1)	
Education (%)	193 (100)	144 (100)	0.559	184 (100)	128 (100)	0.099	143 (100)	153 (100)	–	311 (100)	317 (100)	–
HAVO	73 (37.8)	50 (34.7)		50 (27.2)	46 (35.9)		0 (0)	0 (0)		311 (100)	317 (100)	
VWO	120 (62.2)	94 (65.3)		134 (72.8)	82 (64.1)		143 (100)	153 (100)		0 (0)	0 (0)	
Science	190 (100)	146 (100)	0.719	184 (100)	133 (100)	0.215	142 (100)	150 (100)	0.959	310 (100)	316 (100)	0.024
Nonscience	44 (23.2)	39 (26.7)		107 (58.2)	73 (54.9)		29 (20.4)	31 (20.7)		55 (17.7)	36 (11.4)	
Science	144 (75.8)	105 (71.9)		77 (41.8)	60 (45.1)		113 (79.6)	119 (79.3)		255 (82.3)	280 (88.6)	
Don't know	2 (0.0)	2 (0.0)		0 (0.0)	0 (0.0)		0 (0.0)	0 (0.0)		0 (0.0)	0 (0.0)	

The given percentages have been calculated from the number of students for which data is available for that variable. The percentages have been rounded off to one decimal place. Pearson chi-square was used to calculate differences per country and the control group between pre- and post-test. T = 0 represents the pre-test and T = 1 represents the post-test after 10 weeks. Education represents the level of education, in which HAVO stands for advanced general secondary education and VWO stands for pre-university education level. Science represents the interest of the students, measured by their (preferred) choice of curriculum.

presents the pre- and posttest characteristics of the module participants from all three countries and the control group. In all groups, except the Surinamese group, the age category in the posttest was significantly higher than in the pretest. However, for gender and education, the characteristics did not show any significant differences between the pre- and posttest per country. In Indonesia, the preference for science was significantly higher in the posttest than in the pretest.

On average, in the Netherlands, most of the participating students were male, whereas in Indonesia, they were mostly female. Generally speaking, in the Netherlands, the students from both the control group and the intervention group were significantly younger than average. The pretest showed that on average more students attended preuniversity education in the Netherlands, Suriname, and in the control group. In Indonesia, all students attended advanced general secondary education. In the posttest, the percentage of preuniversity education students in the Dutch intervention group rose to 65.3%. In the control group, this percentage decreased to 64.1%.

In both the pre- and posttest, the amount of participants from the intervention group in the Netherlands and Suriname that chose scientific profiles was not significantly different from the average. The control group consisted of less students with scientific profiles than average and Indonesia had more students with science-related profiles.

### Correlations between knowledge score and attitude and behavior.

Pearson's coefficients showed a positive and significant correlation between the knowledge scores and all four components regarding attitude and behavior (Table 3

**Table 3:** Pearson correlation coefficients between knowledge, attitude, and behavior

		1	2	3	4	5
1	Knowledge	1				
2	Attitude and awareness	0.20**	1			
3	Attitude and risk infection	0.07*	0.18**	1		
4	Behavior and risk infection	0.14**	0.47**	0.20**	1	
5	Behavior and life sciences	0.14**	0.51**	0.15**	0.38**	1

The correlation coefficients shown have been calculated from all values in the intervention groups at both the pre- and posttest. * *P* < 0.05 ** *P* < 0.01 *** *P* < 0.001.

). Knowledge was most strongly correlated with attitude and awareness (*r* = 0.20). Students who scored higher on attitude and awareness also scored higher on behavior regarding risk of infection (*r* = 0.47) and behavior regarding life sciences (*r* = 0.51).

### Knowledge.

**Figure 3.**
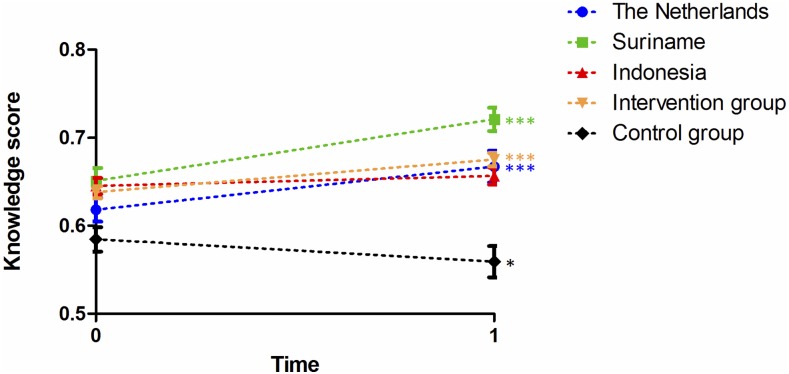
The impact of the Viruskenner on students' knowledge. The knowledge of the participating and nonparticipating students per country before and after the intervention is represented by the mean percentage of the true/false questions in the questionnaire that were answered correctly. The blue line represents the Netherlands, without the control group. The orange line represents all intervention groups, so from the Netherlands, Suriname, and Indonesia. * *P* < 0.05; ** *P* < 0.01; *** *P* < 0.001. This figure appears in color at www.ajtmh.org.

During the project, the answer to one of the 32 knowledge questions changed, due to the MERS epidemic in South Korea. Because of the confusion surrounding this question, we decided to exclude it from the analysis. Analyses per country showed differences in achieved knowledge (Figure 3

), with mean knowledge increasing in all three participating countries. For Suriname and the Netherlands, this increase was significant (*P* < 0.001). The overall effect size (Cohen's d) for all intervention groups was 0.43, which represents a medium effect. At 0.77, the effect size for Suriname was the highest. The effect size for the Netherlands was 0.52, which also represents a large effect.^36^ For example, in the Netherlands, the percentage of correct answers on the statement “Dengue is a virus infection that is transmitted by a tiger mosquito” raised from 71% correct in the pretest to 90% correct in the posttest (the correct answer is true). The score for “If someone is infected with HIV this person has AIDS” raised from 76% to 83% (the correct answer is false because acquired immunodeficiency syndrome is a syndrome in which the immune system is suppressed and opportunistic infections can cause illness, which can be prevented in HIV infected individuals by taking antiretrovirals).Although in some other questions the percentage of correct answers differs only one percentage point between the pre- and posttest. The mean percentage of correct answers on a few questions declined. The mean total knowledge in the control group decreased significantly (*P* = 0.032).

In the multiple regression analyses, the variable participation (control group or intervention group) contributed significantly (*P* < 0.001; B = 0.078) to the knowledge outcome. The variable time point (pre- or posttest), however, did not. Most information about the impact of the module on knowledge is given by the interaction between participation and time point, which was significant (*P* < 0.001; B = 0.053). Other variables that contributed significantly to knowledge were gender, age > 16 years, and the school (Table 4

**Table 4:** Multiple regression analysis

	Knowledge	Attitude and awareness	Attitude and risk infection	Behavior and risk infection	Behavior and life sciences
	B (SE)	B (SE)	B (SE)	B (SE)	B (SE)
Participation	0.078 (0.011)***	−0.170 (0.074)*	−0.012 (0.089)	−0.037 (0.080)	0.264 (0.096)**
Time point	−0.015 (0.011)	−0.380 (0.070)***	0.099 (0.084)	−0.288 (0.076)***	0.139 (0.091)
Participation × time point	0.053 (0.012)***	0.430 (0.078)***	0.113 (0.093)	0.310 (0.084)***	−0.104 (0.101)
Girl	0.012 (0.005)*	0.148 (0.031)***	0.025 (0.037)	0.210 (0.033)***	−0.052 (0.040)
Age < 14	−0.039 (0.063)	−0.004 (0.342)	0.252 (0.408)	−0.097 (0.369)	−0.099 (0.441)
Age 14	0.007 (0.007)	−0.061 (0.049)	−0.047 (0.058)	0.011 (0.052)	−0.098 (0.063)
Age 16	−0.013 (0.007)	−0.019 (0.044)	−0.035 (0.053)	0.161 (0.048)**	0.087 (0.057)
Age > 16	−0.031 (0.011)**	−0.100 (0.069)	−0.092 (0.082)	0.201 (0.074)**	−0.103 (0.089)
School 2 NL	−0.054 (0.013)***	0.225 (0.084)**	−0.091 (0.101)	−0.080 (0.091)	−0.051 (0.109)
School 3 NL	−0.055 (0.020)**	0.173 (0.130)	0.266 (0.155)	−0.127 (0.140)	0.159 (0.168)
School 4 NL	−0.052 (0.016)**	0.030 (0.105)	−0.034 (0.125)	−0.363 (0.114)**	−0.005 (0.136)
Suriname	0.021 (0.012)	1.086 (0.081)***	0.213 (0.096)*	0.421 (0.087)***	0.624 (0.104)***
Indonesia	−0.006 (0.015)	1.120 (0.070)***	−0.016 (0.115)	0.233 (0.105)*	1.135 (0.125)***
VWO	0.019 (0.011)	0.212 (0.070)**	0.153 (0.084)	0.009 (0.076)	0.430 (0.091)***

B = unstandardized coefficient; SE = standard error.

* *P* < 0.05; ** *P* < 0.01; *** *P* < 0.001.

Participation: The control group has a value of 0 and the participants have a value of 1. Time point: the pretest has a value of 0 and the posttest has a value of 1. Participation × time point is the interaction between the two values. For all other variables, participants for whom that variable applies have a value of 1, and the others a 0.

). The mean tolerance of all variables in the regression analyses is 0.3. Although this suggests that there is some multicollinearity between predictors, this value is no reason for concern.^30^

The data from the knowledge quiz showed that the Netherlands had a mean score of 70.7%, with Suriname scoring 59.6%.

### Stigma and fear.

The first question regarding stigma and fear was “I don't want to mix with people who have HIV” and the second one was “I am afraid that I will get infected by Ebola.” Generally speaking, the module participants' answers did not change significantly. However, the results per country showed a significant decrease in Suriname on both questions (*P* = 0.009; effect size [ES] = 0.31 and *P* = 0.001; ES = 0.42, respectively); other countries showed no significant differences in separate analyses, neither did the control group.

### Attitude and behavior regarding virus infections and life sciences.

Figure 4

**Figure 4.**
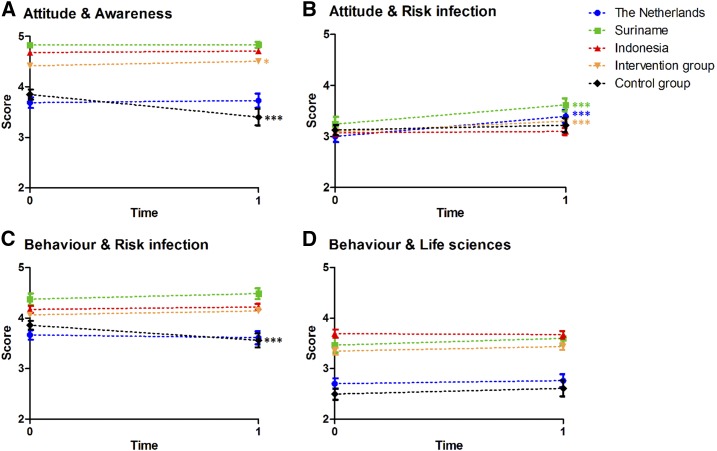
The impact of the education module on students' attitude and behavior. The graphs illustrate the changes per country in the four different components of the attitude and behavior questions that were answered on a 5-point Likert scale. A higher score represents a more positive attitude or healthier behavior. Panel A shows the score for the attitude and aswareness component, panel B for attitude and risk infection, panel C for behavior and risk infection and panel D for behavior and life sciences. The blue line represents the Netherlands, without the control group. The orange line represents all intervention groups, so from the Netherlands, Suriname, and Indonesia. * *P* < 0.05; ** *P* < 0.01; *** *P* < 0.001. This figure appears in color at www.ajtmh.org.

shows the changes in the four components regarding attitude and behavior. In the intervention group (all countries combined), attitude and awareness increased significantly (*P* = 0.028; ES = 0.12) and so did attitude and risk infection (*P* < 0.001; ES = 0.30). Behavior and risk infection increased, but with the chosen *P* value of 0.05, the increase was on the borderline of significance (*P* = 0.062; ES = 0.11). Behavior and life sciences also increased with borderline significance (*P* = 0.060; ES = 0.10). In the control group, attitude and awareness and behavior and risk infection decreased significantly (*P* < 0.001; ES = 0.67 and *P* < 0.001; ES = 0.51, respectively). Attitude and risk infection and behavior and life sciences both showed a slight, but nonsignificant increase.

Although attitude, and even behavior, in the intervention group seemed to increase, in the subanalysis per country, we only found a significant increase in attitude and risk infection for the Netherlands and Suriname (Figure 4).

The multiple regression analysis showed that as main effects, participation and time point both contributed significantly to attitude and awareness. We also found significant interaction between participation and time point for this outcome (*P* < 0.001; B = 0.430). The independent variables gender, school 2, education level, and countries also contributed significantly to attitude and awareness. For the attitude and risk infection outcome, only Surinamese students had higher scores (*P* < 0.05; B = 0.213). For behavior and risk infection, the main variable participation was not significant. But time point was, and it had a negative effect (*P* < 0.001; B = −0.288). The interaction between these two resulted in a significantly positive effect (*P* < 0.001; B = 0.310). Being older, the school and the country also contributed significantly, to behavior and risk infection. Behavior and life sciences were influenced by participation in the module; however, time point had no significant effect and the interaction was not significant either. Countries and education level, however, did contribute significantly to behavior and life sciences (Table 4).

### Appreciation of the project.

Generally speaking, the students enjoyed participating in the education module and said that it taught them a lot about infectious diseases. The score on the statement “I enjoyed working on the project Viruskenner” was measured on a scale from 1 (totally disagree) to 5 (totally agree). The mean score in the Netherlands was 3.2, in Suriname 4.37, and in Indonesia 3.93. In total, 90% of all students that participated gave a score of 3 or higher. Supplemental Table 1 in the supplementary data reports how they answered the other evaluation questions.

### Teacher interviews.

The first and second school participated in the project for 5 and 4 years, respectively, but the head teacher of the first school was involved for the first time. The project was completely new for the third and fourth schools. The first school allowed the most time for students to work on the project, 6 hours a week for 8 weeks. The fourth school allowed 5 hours a week for 8 weeks. The second and third schools allowed 5 and 4 hours a week, respectively, for 6 weeks in each case. In the first three schools, the students had no other lessons about viruses during the project period; only in school four did the biology teacher pay some extra attention to them. None of the teachers let the students prepare for the kick-off, but the teacher in the fourth school told them to read the manual. In the first and fourth schools the students themselves decided on the composition of the collaboration groups and chose their subject of preference. Students in the second school also decided their group composition, but straws were drawn to allocate the subjects. The teacher in the third school divided the students and subjects over the groups randomly. All teachers reported that contact with their coaches during the project was good, although it has to be said that there were some communication problems with the teachers in the second school. And while school number three's teacher said that the contact with the coach was very helpful and amicable, he added that the students got to learn more about the world of scientific research, and that this aspect might have been emphasized even more. Another remark was made by a teacher in the first school, who said that the website should be promoted for learning purposes more frequently and contact with the coaches could be more intensive. All teachers responded positively to the question: “What do you think the students learned from the module?” The teacher of the first school said he thought students are now more aware of the worldwide impact of infectious diseases. He even remarked that during the break on the final day he noticed that more students washed their hands after going to the bathroom. The teacher in the second school insisted that students are now more focused on viruses in the news, such as Ebola or MERS, and that there is a gap between knowing and doing. Finally, a teacher in the fourth school concluded that during the completion of the posttest questionnaires he got the distinct impression that the students learned a lot.

### User data online resources.

Although the education was mainly face to face, online supportive resources were available to increase the educational impact. The graph in S1 shows the use of the website and social media in time.^27^

## Discussion

After adjusting for age, sex, education level, school, and country, Viruskenner proved to be an effective education module for increasing the knowledge of young people in the Netherlands and Suriname of virus infections, according to this nonrandomized intervention study. With all limitations of this study design taken in mind, we describe a positive correlation in knowledge, attitude, and behavior in the participating secondary school students. Participation had a positive effect on attitude and awareness. This effect was higher among females and students who had attained a higher level of education. Knowledge, behavior, and risk infection were higher in female and older students (16+). And while the attitude components increased in the intervention group, the behavior components only showed an increasing trend. There was no significant effect of participation on attitude and risk infection, but there was on behavior and risk infection. This might be explained by the positive effect in the control group for attitude and risk infection but negative for behavior and risk infection. It might be due to there being less motivation in the control group to fill in the questionnaires. The education module had less impact on students' knowledge in Indonesia.

The somewhat limited impact on Indonesian participants could be explained by their lower level of involvement.^37^ All students in the Netherlands and Suriname developed a prevention tool and prepared a presentation. However, the evaluations found that in Indonesia only a selection of the students did. Additionally, in Suriname (and partly in the Netherlands), family members were invited to attend the final day. In Indonesia, this was not possible due to the limited space. The engagement of families could well have had a positive effect. Another striking fact in Indonesia was the relatively high scores for attitude and awareness and behavior, in both pre- and posttests. The same was true for Suriname, which might point to cultural differences with the Netherlands. Collectivistic countries, like Suriname and Indonesia, tend to give more socially desirable answers to questionnaires than individualistic countries like the Netherlands.^38^ Overall, most students of all countries enjoyed working on the project. Although most outcomes in the intervention group showed a positive trend or change, in the control group knowledge, attitude, and awareness and behavior and risk infection decreased. These students did not differ significantly in gender, education level, or profile between pre- and posttest. The decreased outcomes might be explained by reduced motivation in doing the same test twice.

To our knowledge, this is the first study to evaluate an education module on several viruses in several continents. The heterogeneity of the study population increases the external validity of the study. Comparing the results of the same education module in different countries gave insights in the importance of educational factors on the impact. In each country, the pre- and posttests were compared. However, a limitation of the study is that only the Netherlands had a control group. The control group consisted of more students that had chosen a nonscience curriculum than the intervention group. However, there was no significant difference in knowledge score between the nonscience and science students in the control group. Although science students scored higher on attitude and awareness and behavior and life science questions.

Although the time that schools spend on the project differed, no direct relationship was found between the hours spent and the results achieved. Making the results translatable to schools that could participate to the module and would spend at least 4 hours per week during 6 weeks. Due to logistics, randomization of schools was not possible. For a maximum effect, it is important to embed the project in the curriculum, so schools were chosen by a curriculum in which it would fit, as it is in the Netherlands with Technasium. Multiple participating schools per country would have added value as it would have enabled us to perform a proper multilevel analysis, instead of a multiple linear regression analysis. Furthermore, it might be good to measure any balancing measure, for example, the mean grades, to determine whether there are any unanticipated harms to scores on other subjects in school, due to the time the students spent on the project. Although the project is embedded in the curriculum, the harms to other subject would be minimized. The questionnaires were composed with accuracy in Dutch (the national language in the Netherlands as well as in Suriname). The ones that were used in Indonesia were translated to Bahasa Indonesia without back translation. Self-reported questionnaires are useful to measure knowledge changes. However, self-reported attitude and behavior have to be interpreted with caution. The effect was little and could even be due to overestimation. The effect of participation in the module on knowledge, however, was large in two of three countries. Measuring a long-term effect in these countries as well would be of additional value.

A clear effect on knowledge, but a negligible or nonexistent effect on attitude and behavior is common in educational research. Several studies pertaining to HIV or sex education show that knowledge increased after participation in an education module.^10^^,^^39^^,^^40^ We only found a few studies in which peer education did not increase knowledge.^41^^,^^42^ The literature about stigma, awareness, and attitude is inconclusive. Some studies conclude that awareness can be increased or that attitudes can be changed, whereas others conclude that the effects on these is limited.^41^^–^^51^

According to the available literature, behavior is the most difficult part to measure and improve. Most studies about virus education evaluate HIV prevention programs. Condom use or the intention to use condoms are measured most frequently. In self-reported questionnaires these outcomes improved significantly in some studies, which is promising. However, other studies did not find a significant improvement.^10^^,^^41^^,^^45^^,^^47^^,^^52^ We found two studies, both conducted in Africa, that tested behavioral change based on the prevalence of virus infections. In them, participants' blood samples were tested for HIV and herpes simplex virus antibodies, before and 3–8 years after an education module or compared with a control group. However, no significant differences were evident in infection rates.^53^^,^^54^ So what factors play a role in making an educational intervention effective in changing behavior? We found some studies that based their intervention on the Health Promoting School Framework of the WHO proved to be successful in changing health-related behavior.^55^ Important elements of this framework which were applied in these studies were implementation of the intervention in the school curriculum, involvement of the school environment in the project, and involvement of family and society in the intervention.^55^^,^^56^ The Viruskenner education module was implemented in the Technasium curriculum in the Netherlands, but was not part of the curriculum in Suriname and Indonesia. The family and society were involved in the project, particularly in the Netherlands and Suriname. However, stronger involvement of the school environment and ethos in prevention of infectious diseases might increase the impact of the intervention on attitude and behavior in all countries. This might be reached by additional interventions like handwashing posters in the sanitary facilities or selling machines for mosquito nets in the schoolyard, for example. Knowledge that is not translated into behavior change would not make a difference in numbers of virus infections. So adjustments to the Viruskenner module are needed to have a greater impact on attitude and behavior. Active learning has the best chance of being successful if every individual student participates. Students' families have to be closer involved and a sharper focus on infection prevention in school environments is needed. Increasing knowledge is a great first step, because it correlates with attitude and behavior. However, significant improvements in attitude and behavior must be reached to have a possible impact on infection rates. Therefore, further exploration of contributing elements of education modules that reached behavioral changes would be very useful.

## Supplementary Material

Supplemental Table and Figure.
