# Supplementary material for: Experience with a Multinational, Secondary School Education Module with a Focus on Prevention of Virus Infections
Source: Am J Trop Med Hyg. 2017 Apr 24;97(1):97–108. doi: 10.4269/ajtmh.16-0661 (PMC5508890; doi:10.4269/ajtmh.16-0661)
Supplement: Supplementary file 1 [file tpmd160661.SD1.pdf]

Supplemental Table 1
Appreciation questions

Q	Theses	Country	Netherlands		Suriname		Indonesia	
			Mean	SD	Mean	SD	Mean	SD
1	The Opening Day of Viruskenner was interesting.		2.85	1.330	4.16	1.211	4.17	0.855
2	It was clear what we had to do for the project.		3.43	1.244	4.48	0.974	3.86	0.861
3	I enjoyed working on the project Viruskenner.		3.20	1.208	4.37	1.017	3.93	0.844
4	I learnt a lot about infectious diseases during the project.		3.63	1.124	4.43	0.937	4.07	0.868
5	The collaboration in my group was NOT bad.		3.96	1.220	3.74	1.405	3.65	1.090
6	The contact with my coach was good.		2.77	1.134	3.64	1.265	3.56	0.887
7	The Final Day of the project was NOT boring.		2.50	1.298	3.95	1.326	3.35	1.138
8	I think because of the knowledge I gained during the project I have less chance to get a virus infection than I had before the project.		3.06	1.211	3.93	1.159	3.43	0.958
9	I take more precautions to protect myself against virus infections since I participated in the Viruskenner project.		2.61	1.139	4.16	0.990	3.92	0.942
10	It was easy to find someone in Indonesia who could be responsible for our class./My teacher was very enthusiastic about the project.		3.82	1.108	4.66	0.791	4.28	0.806
11	I think the Skype session added value to the project.		–	–	3.34	1.287	3.55	0.956

SD = standard deviation.

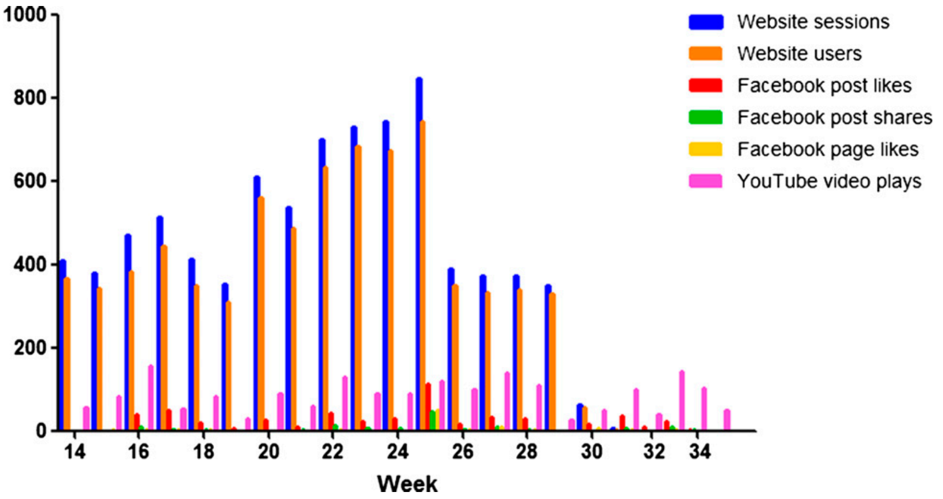

Supplemental Figure 1. The graph shows the activities of users of the website, Facebook page and YouTube channel through the course of the project.
